# De novo assembly and transcriptome characterization of spruce dwarf mistletoe *Arceuthobium sichuanense* uncovers gene expression profiling associated with plant development

**DOI:** 10.1186/s12864-016-3127-y

**Published:** 2016-10-01

**Authors:** Yonglin Wang, Xuewu Li, Weifen Zhou, Tao Li, Chengming Tian

**Affiliations:** 1 The Key Laboratory for Silviculture and Conservation of Ministry of Education, College of Forestry, Beijing Forestry University, Beijing, China; 2 Academy of Forest Inventory and Planning, State Forestry Administration, Beijing, China; 3 Forest Pest Control and Quarantine Station of Qinghai Province, Xining, China; 4 Xianmi Forest Park of Qinghai Province, Menyuan, Qinghai China

**Keywords:** *Arceuthobium sichuanense*, Spruce dwarf mistletoe, De novo assembly, Transcriptome, Development

## Abstract

**Background:**

The parasitic flowering plant dwarf mistletoe (*Arceuthobium* spp., Viscaceae) is one of the most destructive forest pests, posing a major threat to numerous conifer species worldwide. *Arceuthobium sichuanense* (spruce dwarf mistletoe, SDM) infects Qinghai spruce (*Picea crassifolia*) and causes severe damage to spruce forests in Northwest China. SDM is a Chinese native parasitic plant and acquires carbohydrates and mineral nutrition from its hosts. However, underlying molecular basis of the physiological development is largely unknown. Investigations of these physiological traits have been hampered by the lack of genomic resources for this species.

**Results:**

In this study, to investigate the transcriptomic processes underlying physiological traits and development in SDM, we used RNA from four major tissues (i.e., shoots, flowers, fruits, and seeds) for de novo assembly and to annotate the transcriptome of this species. We uncovered the annotated transcriptome and performed whole genome expression profiling to uncover transcriptional dynamics during physiological development, and we identified key gene categories involved in the process of sexual development. The assembled SDM transcriptome reported in this work contains 331,347 assembled transcripts; 226,687 unigenes were functionally annotated by Gene Ontology analysis. RNA-Seq analysis using this reference transcriptome identified 22,641 differentially expressed genes from shoots, flowers, fruits, and seeds. These genes are enriched in processes including organic substance metabolism, cellular metabolism, biosynthesis, and cellular component. In addition, genes related to transport, transcription, hormone biosynthesis and signaling, carbohydrate metabolism, and photosynthesis were differentially expressed between tissues.

**Conclusion:**

This work reveals tissue-specific gene expression patterns and pathways of SDM and implied to a difference between photosynthetic and non-photosynthetic tissues in plants. The data can potentially be used for future investigations on endophytic parasitism and SDM-spruce interaction, and it dramatically increases the available genomic resources for *Arceuthobium* and dwarf mistletoe communities. This preliminary study of the *Arceuthobium* transcriptome provides excellent opportunities for characterizing plant parasitic genes with unknown functions.

**Electronic supplementary material:**

The online version of this article (doi:10.1186/s12864-016-3127-y) contains supplementary material, which is available to authorized users.

## Background

*Arceuthobium*, which are generally referred to as dwarf mistletoes, are obligate heterotrophic plants that parasitize members of Pinaceae and Cupressaceae worldwide [1]. *Arceuthobium* causes the most serious and economically important diseases of conifers worldwide. *Arceuthobium sichuanense* (spruce dwarf mistletoe, SDM) is a unique parasitic flowering plant in China which is considered the most serious vascular parasite on *Picea crassifolia*, *P. purpurea*, *P. likiangensis* var. *balfouriana*, and *P. spinulosa* in Qinghai, Gansu, Sichuan, and Tibet. SDM causes serious mortality in both mature and young spruce trees and has a severe impact on ecological safety in the Sanjiangyuan area of Qinghai province [2]. Despite the great economic and ecological importance of SDM in China, little is known about the basic molecular mechanisms underlying its host plant parasitism mechanism and physiological traits.

**Fig. 1:**
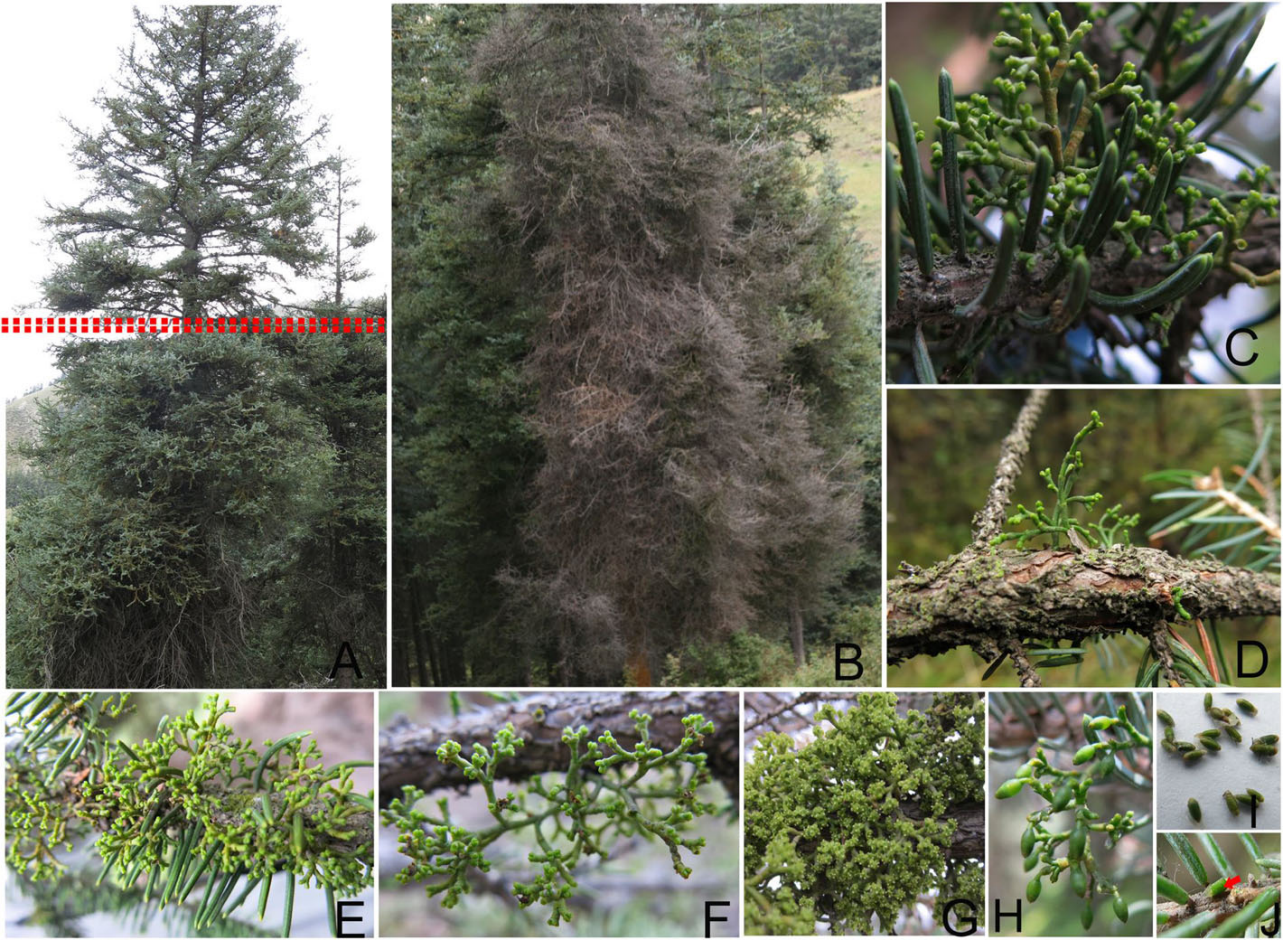
Symptom and morphology of the spruce dwarf mistletoe. **a** Typical symptoms of spruce dwarf mistletoe. Branches at the bottom firstly display witches’ brooms on a mature Qinghai spruce (*double dot lines*). **b** Dieback of branches and death of the entire tree. **c** Spruce dwarf mistletoe parasites on a live stem of a spruce. **d** The swelling of a infected stem. E-I represents shoots (**e**) pistillate flowers (**f**) staminate flowers (**g**) Fruits (**h**) seeds (**i**) of the spruce dwarf mistletoe for collecting samples. **j** A seed attached onto the stem

SDM is a parasite on branches or main stems of spruce trees. Typical symptoms of spruce dwarf mistletoe infection include the development of witches’ brooms, and die-back of individual branches or death of the entire tree in the end (Fig. 1a, b). SDMs usually parasite on stems and branches of spruce (Fig. 1c). Infection can lead to swelling of spruce branches (Fig. 1d). The shoots are 2–6 cm tall and greenish yellow (Fig. 1e). Flowering occurs as early as June and as late as mid-July. Pistillate flowers are 2.5 mm long (Fig. 1f) and staminate flowers are approximately 3.5 mm long (Fig. 1g). Fruits are 1.5–2.0 mm long (Fig. 1h). Seeds were very small and exclusively discharged from mature fruits (Fig. 1i). Discharged seeds attach to the stem or branch of spruce and germinate to infect the host (Fig. 1j, red arrow). The life cycle and infection process of SDM mainly contain flowering, seeds releasing and germination, parasitic shoots development, and endophytic parasitism system [3–5]. Like other dwarf mistletoes, SDM must acquire water and most nutrients from the vascular tissues of the host plant [6]. In *Arceuthobium*, the primary haustorium is a wedge-like projection that penetrates the outer bark extending to the host, and secondary haustoria (“sinkers”) are produced by bark strands that grow radially to the vascular cambium [3]. In addition to *Arceuthobium,* numerous heterotrophic plants acquire water and nutrients via haustoria. In *Cuscuta*, haustoria are developed from the stem of the parasite, penetrating the host tissue and ultimately forming a vascular connection [7]. In addition to allowing transfer of water and nutrients into the parasite, these connections enable the transfer of mRNA, protein, and even pathogens [8–11].

Newly developed genomic and genetic resources will facilitate more rapid progress towards a molecular understanding of plant parasitism [6]. To our knowledge, the molecular and genetic basis underlying the physiologically developmental stages and parasitism in *Arceuthobium* remains unknown. The development of genomic and molecular resources for *Arceuthobium* may lead to the identification of genes vital to its development and parasitism. To date, however, the *Arceuthobium* genome has not been sequenced, and relatively little is known about the molecular basis of physiological development and infection processes by this species.

Next generation sequencing (NGS) is a widely used, powerful technique that provides insights into plant development through genome-wide transcriptome analysis. NGS, accompanied by sophisticated bioinformatics tools for data analysis, including high performance de novo transcriptome assembly, has emerged to facilitate transcriptome analysis in uncharacterized model plants [12–16]. Recently, de novo assembly and analysis of the transcriptomes of parasitic plants have provided important insights into the process of plant parasitism [14, 16–21]. For example, transcriptome analysis of the obligate plant stem parasite dodder (*Cuscuta pentagona*) at diverse tissue and developmental stages shed light on transcriptional dynamics during dodder development and parasitism and the categories of key genes involved in plant parasitism [14].

To better understand the molecular mechanism underlying the morphological transition and parasitism of SDM, it is necessary to first perform detailed transcriptional profiling throughout the development of this parasitic plant. Therefore, to partially fulfill our long-term goals of expanding genomic resources for SDM and uncovering the transcriptomic basis of physiologically developmental stages in this species, we used RNA-seq to generate transcriptome profiles for each of the four developmental stages of this plant to uncover underlying molecular changes. We obtained a fully annotated transcriptome and used this reference to examine the genome-wide differences in gene expression in the stem, flower, berry, and seed tissue. The results of this study provide insights into the future direction of molecular genetic research in SDM.

## Methods

### Experimental site, disease surveys and SDM tissue collection

The experimental site, Xianmi Forest Farm, is located in northeastern of Qinghai province, China, and covers an area of 0.187 M ha. Qinghai spruces (*P. crassifolia*) are the mainly dominant trees accompanied with other arbores, such as *Betula* and *Sabina przewalskii.* The sampling site is the center of the Forest Farm and is 600 m^2^ in size. In order to collect the new-emerging SDM tissues and reduce tissue difference, we chose three infected mature trees with a disease rating of 2–3 in sampling site, f based on Hawksworth’s 6-class dwarf mistletoe rating system: 0 = uninfected, light infection is a rating of 1 to 2, moderate is 3 to 4, and severe is 5 to 6 [22]. Sampling was carried out from early May to early September in 2014. About 3-year-old young shoots, pistillate flowers, developing and immature fruit, and disperse seeds of SDM from the three individual Qinghai spruces were collected, respectively. All tissues were collected external to the host. The samples were quickly cleaned with sterile water and stored in liquid nitrogen until use. In this study, an individual SDM tissue contributed to each sample for RNA extraction and RNA-seq libraries preparation.

### RNA-seq Library preparation and sequencing

Total RNA was extracted from collected tissues (i.e. shoots, flower, fruits and seeds) respectively using Guanidine thiocyanate (Sigma, 50983)-Chloroform (Sigma, 472476) according to the manufacturer’s instructions. 500 mg plant tissues were used and ground in liquid nitrogen to extract total RNA. Total RNA was treated with DNA-free™ DNA Removal Kit (Ambion, AM1906) to remove contaminated Genomic DNA. RNA purity was checked using the NanoPhotometer spectrophotometer (IMPLEN, CA, USA). Before cDNA synthesis, RNA concentration was measured using Qubit RNA Assay kit (Life Technologies, Q32852). RNA integrity was assessed using the RNA Nano 6000 Assay Kit of the Agilent Bioanalyzer 2100 system (Agilent Technologies, CA, USA). The RIN (RNA integrity number) of all the samples was as follows, Stem 1: 6.6; Stem 2: 7.1; Flower: 6.6; Fruit 1: 6.9; Fruit 2: 6.6; Seed 1: 6.9; and Seed 2: 7.2. Therefore, all RNA samples can be used for RNA-seq platform.

Three micrograms of total RNA per sample was used as input material for the RNA sample preparations. RNA-seq libraries were prepared from two biological replicates (except flowers only one library, due to low RNA content retrieved), respectively, using a custom high-throughput method for the Illumina RNA-seq library [23]. The clustering of the index-coded samples was performed on a cBot Cluster Generation System using TruSeq PE Cluster Kit v3-cBot-HS (Illumina) according to the manufacturer’s instructions. These RNA-Seq libraries were sequenced on an Illumina Hiseq 2000 platform at Novogene Bioinformatics Technology Co., Ltd., and 100 bp paired-end reads were generated.

### Preprocessing of Illumina Reads and De novo transcriptome assembly

Raw sequenced reads were processed using Trimmomatic software [24]. In this step, clean reads were obtained by removing raw reads containing adapter sequences, reads containing ploy-N (≥10 %) and low quality (sQ ≤5) reads from raw data. At the same time, Q20, Q30, GC-content and sequence duplication level of the clean data were calculated. All the downstream analyses were based on clean data. All the resultant filtered and trimmed set of high-quality reads for each library was then de novo assembled using the Trinity software package (version r2013-02–25) with min_kmer_cov set to 2 by default and all other parameters set default [25]. To calculate abundance estimation for each unigene, clean data were mapped back onto the assembled transcriptome and read count for each unigene was obtained from the mapping results. To quantify gene expression abundance, FPKM (fragments per kilobase per transcript per million mapped reads) was used, which is made for paired-end RNA-seq and takes into account that two reads can map to one fragment. To avoid false positive estimation of gene expression, unigene with one or more FPKM were retained for downstream analysis.

### Functional annotation of the transcriptome

The assembled unigene from the final transcriptome were annotated by mapping them to several public databases. To assign predicted gene descriptions for the assembled unigenes, they were aligned against were compared with the NCBI nonredundant (NR) database, NCBI nucleotide sequences (NT) database, eukaryotic ortholog groups (KOG) database, KEGG ortholog (KO) database, respectively, using BLASTX with a significance threshold of E-value ≤10^−5^. Unigenes were also compared against the UniProt database and protein family (PFAM) database using default parameters, respectively.

The Gene Ontology (GO) terms describing biological processes, molecular functions, and cellular components for functional categorization were analyzed using Blast2go software [26]. The E-value filter for GO annotation was 1e^−6^. The pathway assignments were carried out by sequence searches against the KO database, also using the BLASTX algorithm with an E-value threshold of 10^−5^. After the processes, proper GO terms and KO pathway were generated.

### Differential expression analysis

RNA-seq by expectation maximization (RSEM), which allows for an assessment of transcript abundances based on the mapping of RNA-seq reads to the assembled transcriptome, was used for transcript abundance estimation of the de novo-assembled transcripts [27]. Differential expression analysis of two groups was performed using the DEGseq R package (1.10.1) [28]. DEGseq provides statistical routines for determining differential expression in digital gene expression data using a model based on the negative binomial distribution. The resulting P values were adjusted using false discovery rate (≤(n d). In this analysis, to avoid false positive estimation of differential expression, the unigenes with a threshold P-value <0.001 and the absolute value of log2Ratio (fold change) >1 screened by DEGseq were assigned as differentially expressed genes. Pearson correlation coefficient was calculated among the seven samples according to genes’ expression profiles. R package was used for visualization of results and read dispersion.

GO enrichment analysis of the differentially expressed genes (DEGs) was implemented by the GOseq R packages based on Wallenius non-central hyper-geometric distribution [29]. We calculated the numbers of all DEGs, up regulated and down regulated genes to each GO term, respectively. As for KEGG enrichment analysis of the DEGs, we used KOBAS software to test the statistical enrichment of DEGs in KEGG pathways. Rich factor was used to represents enrichment intensiveness, which means that the ratio of the DEGs number and the number of genes have been annotated in this pathway [30].

### RT-PCR analysis

We used PCR to validate the assembled transcriptome from the RNA-seq experiment. RNA extracted from shoots, flowers, fruits and seeds using Guanidine thiocyanate-Chloroform protocol. Total RNA was treated with DNase I (Invitrogen, 18068–015). Complementary DNA from total RNA was prepared using the SuperScript III First-Strand Synthesis System for RT-PCR (Invitrogen, 18080400) according to the manufacturer’s protocol. To confirm the presence of predicted unigenes, one microliter of the complementary DNA was used for amplification by PCR using the primers listed in Additional file 1: Table S1. The qRT-PCR was carried out using SYBR green (SuperReal Premix Plus; TIANGEN, China) methodology and the ABI 7500 real-time PCR system (Applied Biosystems, USA). The rDNA gene (c78229_g2) was used as internal reference for all the qPCR analyses. Analyses of each gene were conducted in quadruplicate. The specificity of qRT-PCR primers was confirmed by melting curve analyses. Relative gene expression was calculated according to the △△CT method. The qRT-PCR results were obtained from two biological replicates and four technical repeats for each gene and sample.

## Results

### Illumina sequencing and de novo assembly

To obtain a global, comprehensive overview of the spruce dwarf mistletoe transcriptome, RNA was extracted from four different tissues including shoots, flowers, fruit, and seeds. A total of 468,114,690 paired-end reads (100 bp) were obtained from the four tissue samples on the Illumina HiSeq2000 platform. After preprocessing and filtering of reads (involving the removal of low-quality sequences), a total of 456,774,708 high quality paired-end reads were generated (Table 1) and subjected to transcriptome assembly using the Trinity software package [25]. Using overlapping information from high quality reads, 331,347 transcripts and 226,687 assembled unigenes were generated, which counted for 228,836,465 and 120,878,370 bp, respectively (Table 2).

**Table 1:** RNA-seq data statistics

Samples^a^	Raw reads	Clean reads^b^	Q20(%)^c^	Q30(%)^d^	Mapped reads
Stem 1	59,102,236	57,577,862	93.85	88.22	47,226,724^e^ (82.02 %)^f^
Stem 2	67,819,362	66,055,988	94.11	88.62	53,359,164 (80.78 %)
Flower	67,947,254	66,335,064	93.72	88.03	53,283,472 (80.32 %)
Fruit 1	75,874,632	74,068,030	93.49	87.66	60,369,578 (81.51 %)
Fruit 2	68,449,270	66,996,440	93.57	87.79	54,771,500 (81.75 %)
Seed 1	69,280,068	67,642,194	93.84	88.16	53,799,756 (79.54 %)
Seed 2	59,641,868	58,099,130	93.58	87.7	46,526,234 (80.08 %)
Total reads	468,114,690	456,774,708			

^a^1 and 2 represent two independent biological replicates

^b^The number of reads generated from sequencing after filtering low quality reads (Q ≤ 5)

^c^Q20: The percentage of bases with a Phred value >20

^d^Q30: The percentage of bases with a Phred value >30

^e^The number of reads from clean data that were mapped back onto the assembled transcriptome

^f^The percentages of reads account for the mapped reads

**Table 2:** Summary of de novo assembly of transcriptome

	Transcripts	Unigenes
Total number	331,347	226,687
Average length (bp)	691	533
Minimum length (bp)	201	201
Maximum length (bp)	24,940	24,940
Number, ≤500 bp	201,559	164,554
Number, >500 bp	129,788	62,133
N50^a^ (bp)	1098	690
N90^b^ (bp)	273	237
Total nucleotides	228,836,465	120,878,370

^a^N50 is defined as the length of the largest contig from all the contigs ranked smallest to largest that represents 50 % of the assembly lengthy

^b^N90 is defined as the length of the smallest transcript in the sorted list of all transcripts where the cumulative length from the largest transcript to the smallest transcript is at least 90 % of the total length

**Fig. 2:**
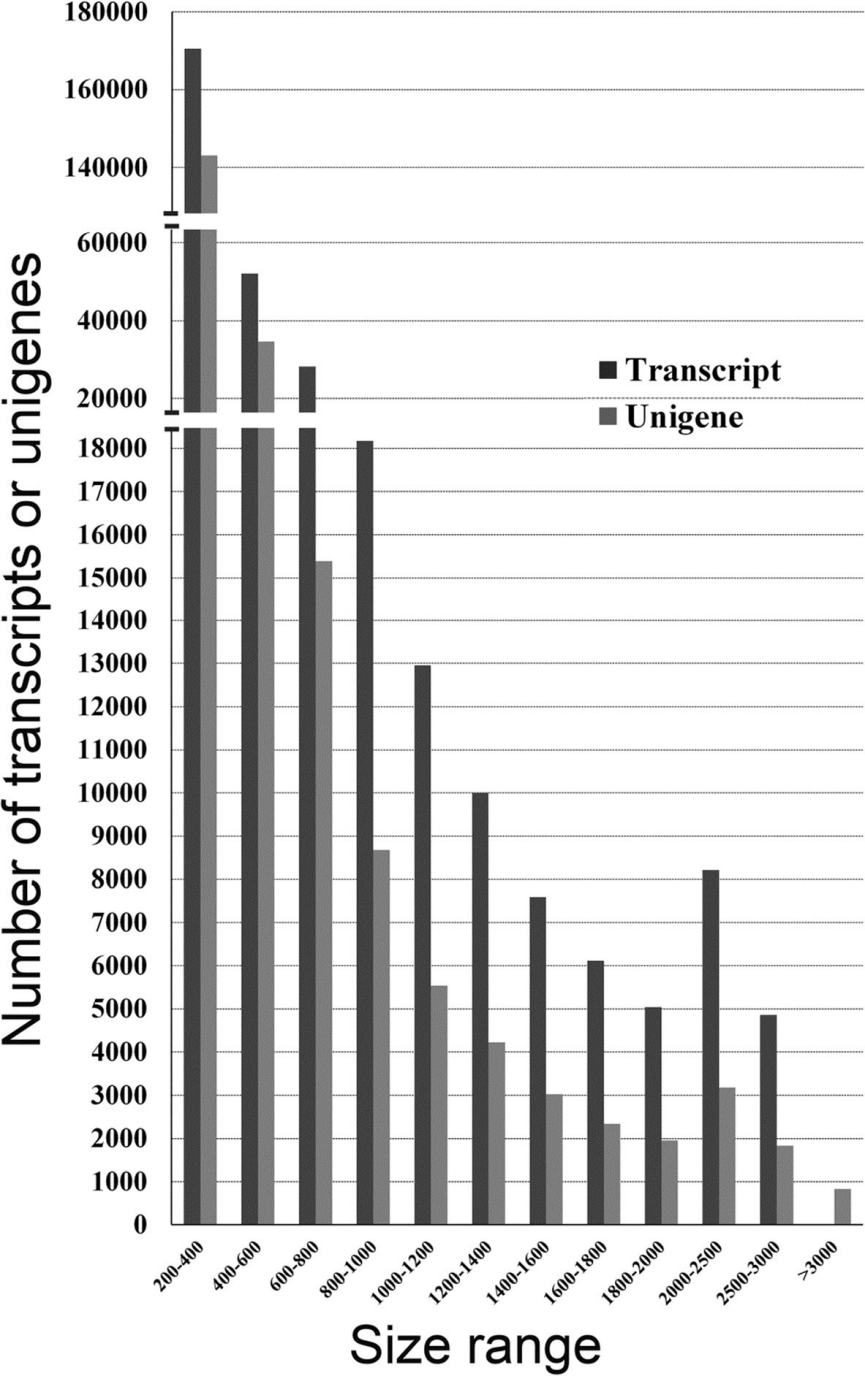
Unigene and transcript size distribution. Unigene and transcript size distribution showing the high proportion of small-sized transcripts in SDM assembled transcriptome

Over 79.54 % of reads from each sample could be mapped back to the assembled transcripts (Table 1). Approximately 60.8 % of the assembled transcripts were ≤500 bp and 39.2 % were >500 bp; 72.6 % of the unigenes were ≤500 bp and 27.4 % were >500 bp (Table 2). The average length of the transcripts and unigenes was 693 and 533 bp, respectively, with maximum and minimum lengths of 201 and 24,940 bp, respectively. The length distribution of the transcripts and unigenes is shown in Fig. 2. To detect the presence of predicted unigenes, the selected unigenes were amplified by reverse transcription (RT)-PCR and sequenced. Sequencing of the RT-PCR products confirmed that these sequences represent genuine SDM transcripts (Additional file 2: Figure S1).

### Functional annotation of the reference transcriptome

All assembled unigenes were aligned against the public databases including NR database, NT database, Pfam, Swiss-Prot, GO, KEGG and KOG. The number of unigenes annotated by each database is summarized in Table 3. Of the 226,687 assembled unigenes, 87,646 (38.66 %) exhibited sequence similarity to a sequence within the NR database; 101,075 (44.58 %) unigenes were annotated in at least one database; 8,744 (3.85 %) unigenes were annotated in all above databases (Table 3). However, the majority of the unigenes (55.42 %) could not be identified, which is common in de novo sequencing studies [14, 31, 32]. For example, in the desert tree *Haloxylon ammodendron*, approximately 62.48 % of the unigenes are unidentified.

**Table 3:** Statistics of annotation results for A. sichuanense unigenes

NR	NT	KO	UniProt	Pfam	GO	KOG	All database	>1 database
87,646	20,524	29,915	54,552	59,166	64,128	34,148	8,744	101,075

*NR* NCBI nonredundant database

*NT* NCBI nucleotide sequences database

*KO* KEGG Ortholog database

*UniProt* Swiss-Prot protein database

*Pfam* protein family database

*GO* Gene Ontology

KOG, eukaryotic ortholog groups database

**Fig. 3:**
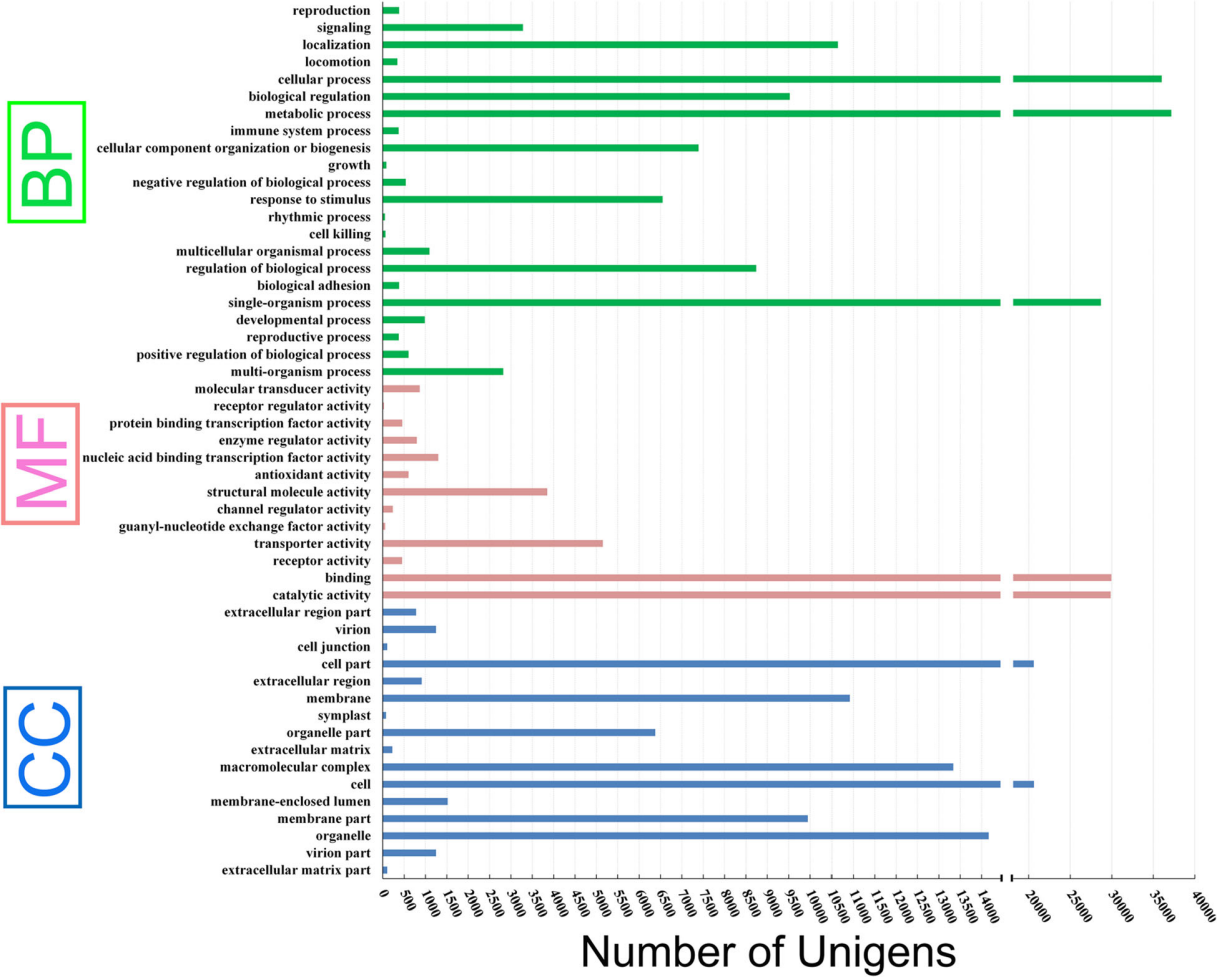
Blast2GO annotation of the SDM transcriptome. GO category distribution of SDM unigenes among level 1 GO categories: biological process (BP), molecular function (MF), and cellular component (CC)

We performed GO functional analysis of the unigenes according to NR annotation. Among the 226,687 unigenes, 64,128 were assigned to the biological process (GO:0008150), molecular function (GO:0003674), and cellular component (GO:0005575) categories; these unigenes were assigned to 52 GO terms. The terms “cellular process”, “metabolic process”, “signal organism process”, “binding”, “catalytic activity”, “cell part”, “cell”, and “cell organelle” were the most highly represented (Fig. 3).

**Fig. 4:**
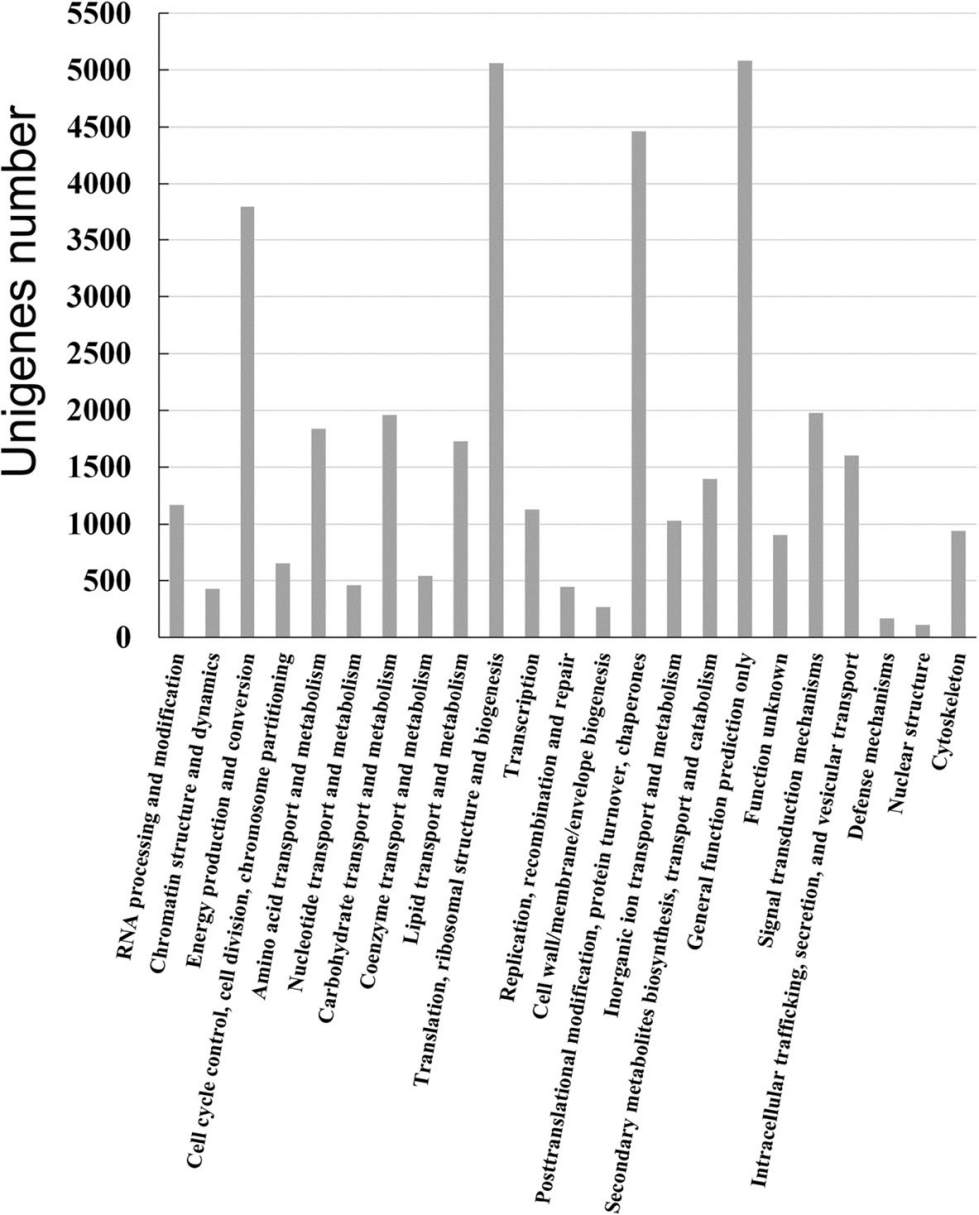
KOG annotation of putative proteins. The unigenes were aligned to the KOG database to predict and classify their possible functions. Of the 226,687 unigenes, 34,148 were annotated and separated into 22 clusters

To further evaluate the reliability of our transcriptome results and the effectiveness of our annotation process, we searched the annotated sequences for genes with KOG classifications. KOG protein database was generated by comparing predicted and known proteins in all completely sequenced eukaryotic genomes to infer sets of orthologs. In this study, 34,148 unigenes were classified into 22 KOG categories (Fig. 4). The largest group was “General function prediction only”, followed by groups such as “Translation, ribosomal structure and biogenesis”, “Posttranslational modification, protein turnover, chaperones”, “Energy production and conversion”, “Signal transduction mechanisms”, “Carbohydrate transport and metabolism”, “Amino acid transport and metabolism”, “Lipid transport and metabolism”, “Intracellular trafficking, secretion, and vesicular transport”, “Secondary metabolites biosynthesis, transport and catabolism” and so on.

### Tissue-specific transcriptome analysis and identification of differentially expressed genes in SDM

**Fig. 5:**
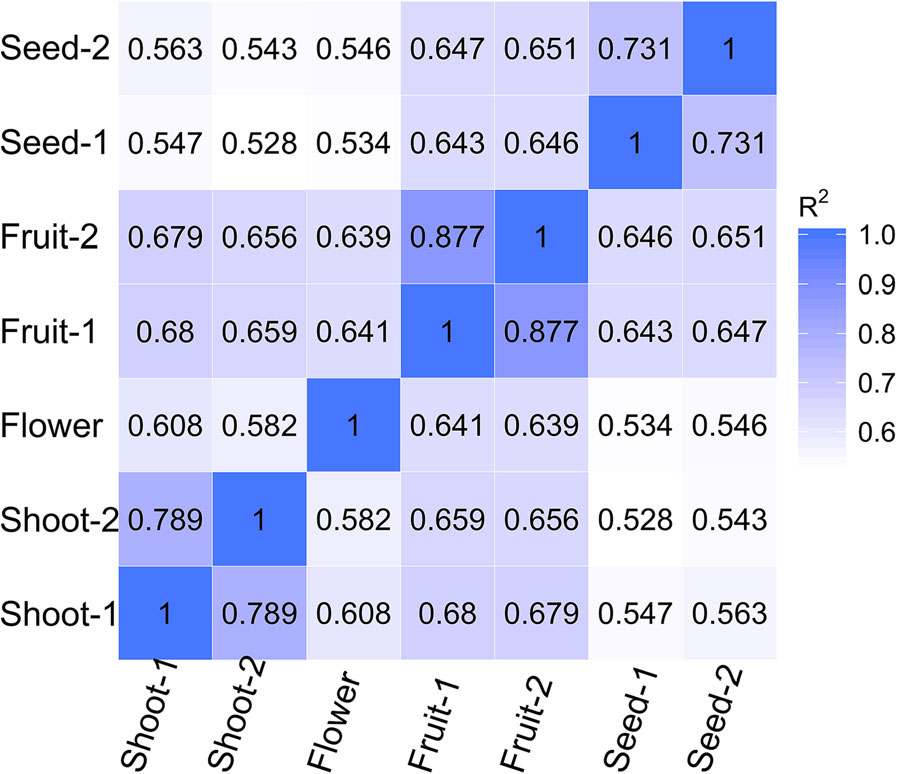
Correlation of expression patterns among the seven RNA-seq libraries. Correlation matrix of the entire dataset. The analysis was performed by comparing the values of the entire transcriptome (226,687) in all seven samples. Correlation analysis was performed using R software

We used the RNA-Seq data to assess differences in the expression of genes in different tissues of SDM i.e., the shoots, flowers, fruits, and seeds in this study. We calculated FPKM values to quantify the expression levels of all unigenes. We aligned Illumina reads from the four developmental stages to the assembled transcriptome and examined the distribution of gene expression values among developmental stages (Table 4). We then comparatively analyzed unigenes with an FPKM value ≥1 in each sample among shoots, flowers, fruits, and seeds. Specifically, we generated a Pearson’s distance correlation matrix to compare the transcriptomes from each sample. The correlation dendrogram illustrates the global, relative relationships among the four tissues (Fig. 5). The correlations of gene expression levels between two biological replicates were high, with an average coefficient (R^2^) of 0.789, 0.877, and 0.731 for shoots, fruits, and seeds, respectively (Fig. 5). No replicates were available for flower tissue due to the small quantity of available RNA.

**Table 4:** Distribution of gene expression values among developmental stages examined

FPKM^a^ Interval	Stem 1	Stem 2	Flower	Fruit 1	Fruit 2	Seed 1	Seed 2
>0	95,864^b^ (42.29%^c^)	105,127 (46.38 %)	111,941 (49.38 %)	94,042 (41.49 %)	90,498 (39.92 %)	118,277 (52.18 %)	106,490 (46.98 %)
0–0.5	31,086 (13.71 %)	33,086 (14.60 %)	34,347 (15.15 %)	33,584 (14.82 %)	32,016 (14.12 %)	38,156 (16.83 %)	36,132 (15.94 %)
0.5–1	20,414 (9.01 %)	22,635 (9.99 %)	23,670 (10.44 %)	18,270 (8.06 %)	17,081 (7.54 %)	24,755 (10.92 %)	21,165 (9.34 %)
1–10	33,042 (14.58 %)	37,831 (16.69 %)	41,853 (18.46 %)	30,520 (13.46 %)	29,857 (13.17 %)	44,341 (19.56 %)	38,641 (17.05 %)
>10	11,322 (4.99 %)	11,575 (5.11 %)	12,071 (5.32 %)	11,668 (5.15 %)	11,544 (5.09 %)	11,025 (4.86 %)	10,552 (4.65 %)

^a^FPKM, fragments per kilobase of transcript per million mapped fragments

^b^The number of genes mapped to the assembled transcriptome

^c^The percentages of genes accounting for all assembled unigenes

**Fig. 6:**
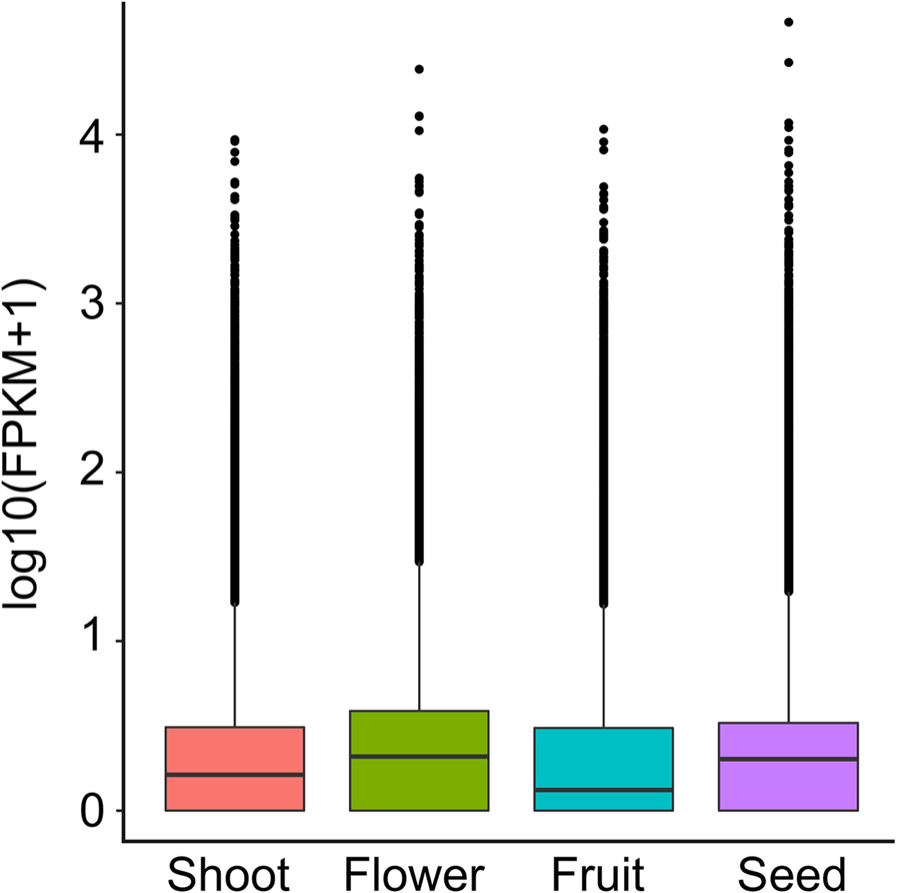
Boxplot of FPKM distribution among the four selected developmental stages. The FPKM boxplot shows the distribution of gene expression levels. The x-axis indicates developmental stages and the y-axis represents the value of log10(FPKM + 1). Each boxplot shows that the maximum, minimum, and median FPKM values across the libraries being compared are comparable

**Fig. 7:**
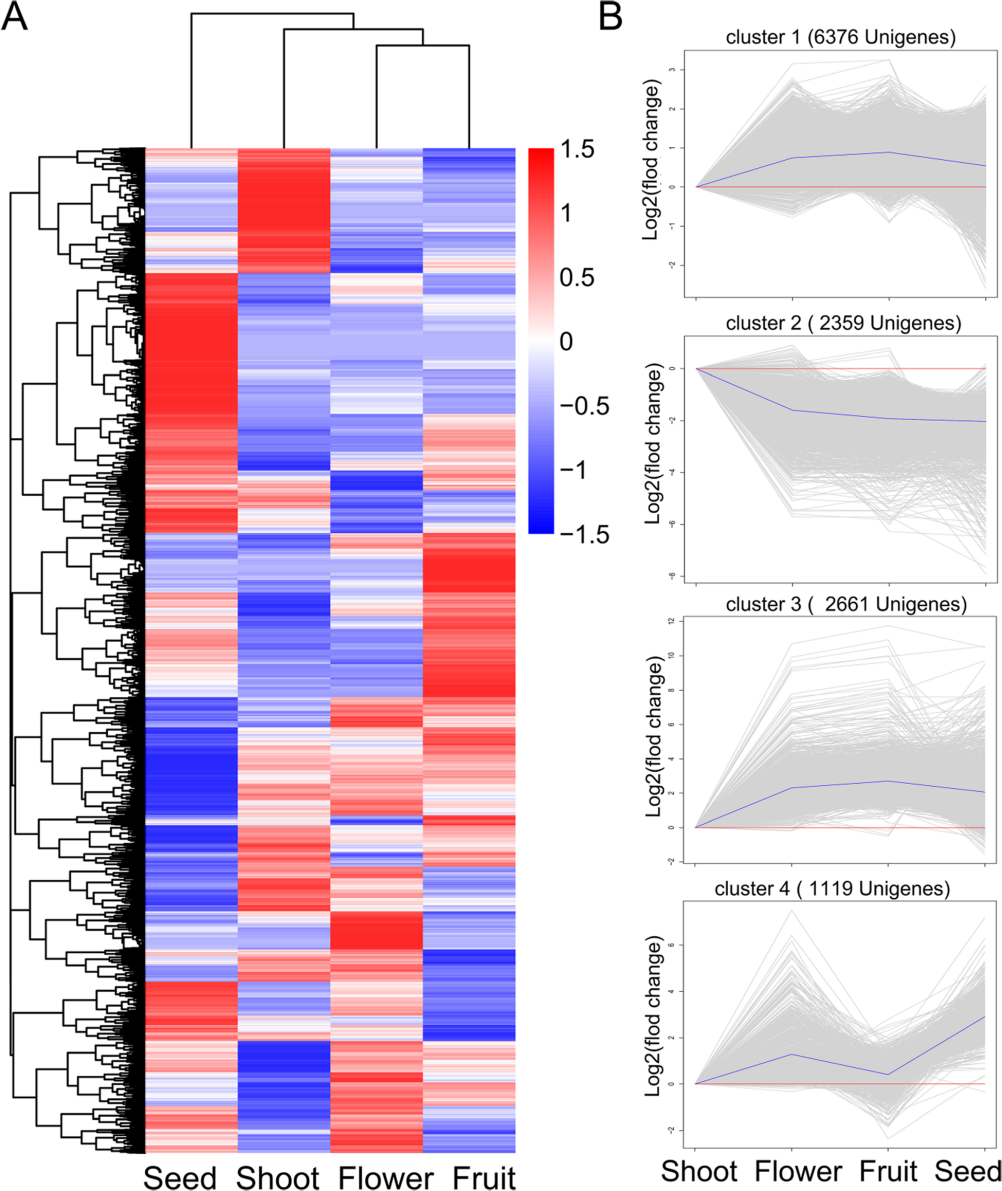
Hierarchical cluster analysis of transcript expression profiles at four selected developmental stages. **a** Cluster analysis was performed using transcriptomic data from shoots, flowers, fruits, and seeds. The log2 of FPKM for each gene was used for hierarchical analysis of the heat map at each of four developmental stages. *Red* and *blue* represent pairwise distances among transcripts above or below, respectively, the mean (*white*) across all four tissues. The heat map represents cluster analysis of 22,641 differentially expressed unigenes according to gene expression level. Expression levels were measured as RPKM from normalized values. **b** Magnified regions of four clusters of interest (I, II, III, and IV) based on certain patterns of tissue-specific expression showing cluster numbers, with the number of associated unigenes in parentheses. The *blue line* on each plot represents the mean expression profile for the cluster, the *red line* represent the baseline

We examined tissue-specific expression patterns using an empirical cutoff value for positively expressed genes. The boxplot distribution of FPKM shows the median and quartile values of differential gene expression among samples (Fig. 6). Furthermore, to investigate gene expression dynamics across different stages of SDM, we performed the hierarchical cluster analysis using normalized read counts. All 25,055 differentially expressed unigenes were used in hierarchical clustering analysis of transcript abundance across the four tissue types. The clustering analysis suggested that flowers showed similar transcriptome profiles to fruits, and seeds showed the greatest difference to other tissues (Fig. 7a). The hierarchical cluster also revealed four main clusters (Fig. 7b). Of the four major clusters, only one (cluster II, containing 2359 unigenes) corresponds to a set of genes that were upregulated in shoot and downregulated in flowers, fruits, and seeds. The three other major clusters represent transcripts upregulated in flowers, fruits, and seeds compared with shoots (Fig. 7b).

**Fig. 8:**
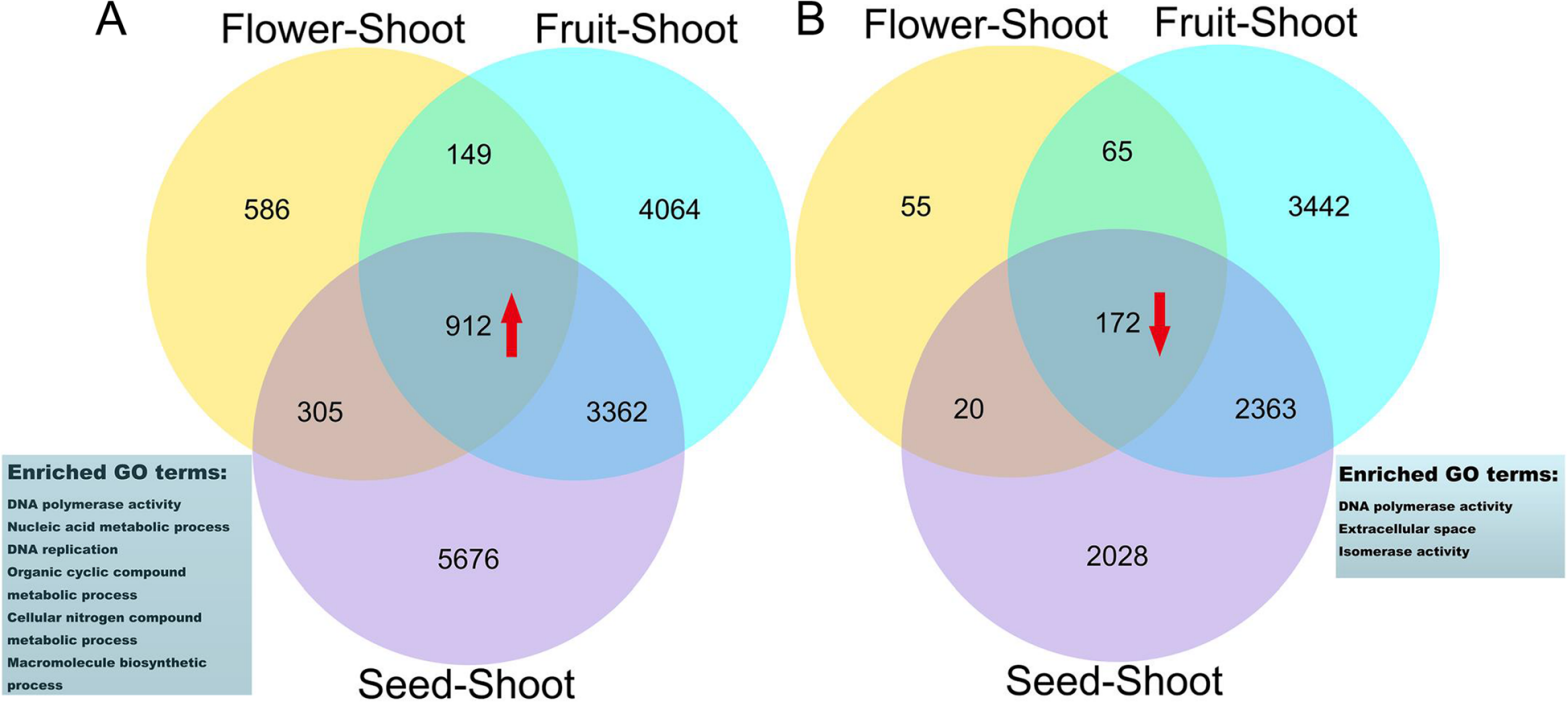
Venn diagram for differentially expressed unigenes and enriched GO terms in developmental stages of SDM*.* Number of unigenes up-regulated (**a**) and down-regulated (**b**) at the stage flower, fruit and seed compared with the shoot, respectively. Overlapping sets of upregulated or downregulated unigenes (labeled by asterisck) among three pairwise comparisons are shown in the Venn diagram. The enriched GO terms of the overlapping unigenes are indicated in the boxes at the bottom, respectively

Differential expression analysis showed that 22,641 unigenes were significantly expressed at shoots, flowers, fruits, and seeds (Additional file 3: Table S2). We constructed a Venn diagram showing the numbers of significantly differentially expressed genes (Fig. 8). We identified 912 upregulated unigenes and 172 downregulated unigenes in the reproductive stages (flowers, fruits, and seeds) compared with the vegetative stage (shoots) (Additional file 4: Table S3). Enriched GO terms of upregulated unigene in reproductive stages were DNA polymerase activity, Nucleic acid metabolic process, DNA replication, Organic cyclic compound metabolic process, Cellular nitrogen compound metabolic process, and Macromolecule biosynthetic process, while downregulated unigenes were enriched in GO terms including DNA polymerase activity, Extracellular space and Isomerase activity (Fig. 8).

In order to verify the expression patterns of DEGs involved in shoots, flowers, fruits, and seeds of SDM, five genes were randomly selected for quantitative real-time PCR analysis. As shown in Additional file 5: Figure S2, these results confirmed the accuracy of our transcriptome profiling.

### Important gene families and metabolic pathways among different tissues in SDM

To gain insight into the physiological and molecular factors underlying the development of SDM, we focused on several gene families based on GO enrichment analysis of differentially expressed genes, including transcription factor, protein kinase, transporter, carbohydrate metabolism, and plant hormone-associated genes. We identified 575 unigenes encoding putative transcription factors, including members of the MADS, WRKY, MYB, and Zinc Finger families (Additional file 6: Table S4), 3654 encoding protein kinases (Additional file 7: Table S5), 3922 encoding transporters (Additional file 8: Table S6), and 434 plant hormone-associated genes (Additional file 9: Table S7). Expression profiles of biogenesis, signal transduction processes, and gene families such as transcription factors, transporters and plant hormone associated genes showed differential expression pattern during development (Additional file 10: Figure S3). Among 434 unigenes involved in hormone metabolism and signal transduction pathways, 59 unigenes involved in plant hormone biosynthesis and signaling were enriched during fruit development: auxin influx carrier and BR-signaling kinase genes were upregulated, whereas unigenes encoding ethylene receptor and ethylene-responsive transcription factor were downregulated. Furthermore, 2668 differentially expressed unigenes involved in carbohydrate metabolism were identified (Additional file 11: Table S8). We have identified 207 differentially expressed unigenes related to photosynthesis during the physiological development (Additional file 12: Table S9).

Many differentially expressed genes were highly enriched in primary metabolism categories, including organic cyclic compound biosynthetic process, aromatic compound biosynthetic process, and nitrogen compound metabolic process. Three unigenes (c209227, c40695, and c54155) involved in energy processes were also differentially expressed, including unigenes homologous to ATP-binding protein EcfA and cytochrome C oxidase subunit genes (Additional file 11: Table S8). We also identified a total of 207 unigenes related to photosynthesis process that was differentially expressed. These unigenes were enriched in cellular component of chloroplast thylakoid membrane (Additional file 12: Table S9).

We compared differentially expressed unigenes in flowers, fruits, and seeds versus the reference tissue (shoots). Transcriptome analysis revealed that 586 and 55 unigenes in flowers, 4064 and 3442 unigenes in fruits, and 5676 and 2028 unigenes in seeds were specifically upregulated and downregulated compared with those in shoots, respectively (Fig. 8). Enriched GO categories of upregulated transcripts in a pairwise comparison of fruits and shoots included RNA-directed DNA polymerase activity, RNA-dependent DNA replication and DNA polymerase activity, metabolic process, cellular component biogenesis, cellular metabolic process, biosynthetic process, and organic substance metabolic process. Unigenes under these enriched GO terms included unigenes encoding NAC transcription factor, lipid transfer protein, heat shock protein, fatty acid hydroxylase superfamily, disease resistance response, Cytochrome P450, and protease inhibitor (Additional file 13: Table S10).

KEGG pathway analysis showed that the differentially expressed genes in stages of flowers, fruits, and seeds compared with shoots were significantly enriched in several pathways, such as Plant hormone signal transduction, Cutin, suberin, and wax biosynthesis, Flavonoid biosynthesis, Phenylpropanoid biosynthesis, Diterpenoid biosynthesis, and Stilbenoid, diarylheptanoid, and gingerol biosynthesis (Table 5). Unigenes encoding auxin influx carrier and BR-signaling kinase were upregulated, whereas unigenes encoding ethylene receptors and ethylene-responsive transcription factors were downregulated. Some of these pathways are completely or highly overlapping and actually belong to two pathways: Plant hormone signal transduction and Cutin, suberin, and wax biosynthesis. For example, approximately 59 unigenes associated with plant hormone biosynthesis and signaling were significantly enriched in fruits (Table 5). These results suggest that these genes play important roles in the physiological development of SDM.

**Table 5:** Top 5 enriched KEGG pathways among pairwise comparisons of flower, fruit, seed with shoots

Comparison	Pathway terms	Rich factor^a^	q-value^b^	No.^c^
Fruit vs shoot	Plant hormone signal transduction	0.44	3.06E^−20^	59
	Terpenoid backbone biosynthesis	0.19	0.0019	28
	Cutin, suberine and wax biosynthesis	0.51	2.31E^−05^	15
	Brassinosteroid biosynthesis	0.70	0.0052	7
	Flavonoid biosynthesis	0.2	3.73E^−08^	7
Flower vs shoot	Phenylpropanoid biosynthesis	0.031	4.18E^−05^	10
	Diterpenoid biosynthesis	0.25	4.18E^−05^	4
	Stilbenoid, diarylheptanoid and gingerol biosynthesis	0.145	0.00019	4
	Pentose and glucuronate interconversions	0.02	0.00065	10
	Plant hormone signal transduction	0.42	4.06E^−14^	56
Seeds vs shoot	Cutin, suberine and wax biosynthesis	0.62	4.45E^−06^	18
	Homologous recombination	0.3	0.010	18
	Carotenoid biosynthesis	0.37	0.012	13
	Stilbenoid, diarylheptanoid and gingerol biosynthesis	0.40	0.014	11

^a^Rich factor means that the ratio of the DEGs number and the number of genes have been annotated in this pathway. The greater of the Rich factor, the greater the degree of enrichment

^b^The q-value was calculated using hypergeometric test through Bonferroni Correction. Q value is corrected p value ranging from 0–1, and less Q value means greater intensiveness

^c^Unigene number in each pathway

## Discussion

*A. sichuanense*, a Chinese endemic parasitic plant which parasitizes the Qinghai spruce *P. crassifolia*, is considered to be the most serious parasite of conifers in China. To our knowledge, the current study provides the first transcriptomic view of *A. sichuanense* and preliminary reports on identification of genes and gene categories underlying its physiological development of the species. Furthermore, we performed comparative transcriptomics during physiological development, finding that 912 and 172 unigenes were specifically up- and downregulated, respectively, in the reproductive stages (flowers, fruits, and seeds) compared with shoots. Functional enrichment of these differentially expressed genes provided clues about the molecular basis of reproduction in SDM.

### Transcriptome assembly and annotation

Great advances in NGS technologies and data mining platforms have led to rapid progress in comparative transcriptomes of non-model, non-crop plants such as parasitic plants [18, 33]. The primary objective of this study was to construct a de novo transcriptome assembly. Here, we carried out paired - end sequencing of RNA-Seq libraries prepared from mRNA isolated from four developmental tissues (shoots, flowers, fruits, and seeds). High throughput sequencing generated more than 456 million filtered reads. Subsequently, resultant transcriptome assembly produced a dataset of approximately 331,347 transcripts, including 226,687 unigenes. More than 101,075 unigenes were annotated by BLAST analysis and mapped to at least one GO category. The annotations provide a resource for further investigating the processes and pathways involved in the development of dwarf mistletoe (Fig. 3). Using the same strategies, Leslie and Baucom [13] constructed the transcriptome of the agricultural weed *Ipomoea purpurea* to assess potential differences in gene expression between herbicide resistant and susceptible lines. Further transcriptomic, genomic analyses will enable parasitic plant gene sequences to be cloned and characterized, which was undertaken to begin elucidating of the genetics and biochemical processes underpinning parasitism and development of parasitic plants and may yield insight into control strategies to combat parasitic plants of economic importance.

### Genes and gene categories associated with plant development

We assessed the function of tissue-specific transcripts of SDM and found that transcripts associated with development primarily encode transporters, protein kinases, and transcription factors, suggesting the strong involvement of transcriptional activation and transport in fruit and seed formation. Similar conclusions can be drawn in previous studies as below. In Arabidopsis, many fruit-specific transcripts encode transporters, including several ABC transporters, some of which may be required for the transport of substrates such as sporopollenin monomers from the tapetum to microspores [34]. In tomato fruit development, transcriptome profiling revealed tissues-specific genes involved in energy metabolism, source-sink relationships, secondary metabolite production [35].

Transcripts of secondary metabolite-related genes in the flavonol biosynthesis pathway (7 unigenes) were substantially enriched at the SDM flowering stage, together with a strong representation of cutin, suberin, and wax biosynthesis genes (15 unigenes). This result is consistent with the finding that, in tomato fruit, the transcripts of flavonol biosynthesis pathway genes are substantially greater in the outer epidermis of fruit, whereas genes associated with cuticle biosynthesis are not specifically expressed in this tissue [35].

Although our current transcriptomic data from shoots, flowers, fruits and seeds are not directly related to the parasitic nature of the plant, carbohydrate metabolism undergoes influence of the parasitism. In transcriptomes of other parasitic plants , such as dodder and Agapanthus praecox ssp. Orientalis, carbohydrate metabolism is related with parasitism [14, 36]. In addition, many studies have examined carbohydrate- and energy-related metabolic pathways during plant development at the transcriptional level. During early fruit development, active sinks such as the growing shoots and fruit compete for limited carbohydrate and nutrient resources [37]. We identified 2668 unigenes involved in carbohydrate metabolism that were differentially expressed during reproductive development in SDM, and found that more unigenes are involved in the starch and sucrose metabolism pathways than in other carbohydrate metabolism pathways that are active in the fruit indicating that these genes play an important role in fruit maturation. Fruit setting and development are highly dependent on the carbohydrate supply. Carbohydrate metabolism, especially starch and sucrose metabolism, contribute to plant fruit development and maturation. These results are consistent with the findings in Siraitia grosvenorii [38], Chinese bayberry [39], *Litchi chinensis* Sonn [40]. In addition, we found that SDM unigenes that participate in energy processes, i.e., ATP-binding protein and cytochrome c oxidase subunit gene, were significantly upregulated in fruit formation of SDM.

We have not found any implication of differentially expressed photosynthesis-related genes on parasitism yet. Here, we noticed that at least gene profiles of plant hormone-associated genes have difference from non-parasitic plants regarding flower and seed development (Fig. 7; Table 5)). Taken together, the SDM reference transcriptome assembled in this study is comprehensive, accurate and useful for future genetic research of SDM.

### Shortcomings of the current study and future directions

The current study reports the first developmental transcriptome from a dwarf mistletoe species, *Arceuthobium sichuanense*, yet there remain major gaps in our understanding of the molecular bases that trigger and regulate endophytic system formation, especially haustorial formation and development. In our laboratory, using paraffin section and light microscope, we found that the cortical strands of the entophytic system of SDM extended by squeezing the cortex cells of the host and developed aerial shoots and sinkers. The ultrastructure of SDM haustoria is underway as well. One of our future objectives is to characterize the transcriptomes of haustorial formation and development of SDM. Combined with the transcriptomes at the *Arceuthobium*–spruce interface, the developmental transcriptomes of *Arceuthobium* obtained in the current study will be useful for investigating the fundamental aspects of the development, biology, and parasitism of parasitic plants.

## Conclusion

SDM is a devastating pest of spruce, posing a serious threat to the ecological value of the Sanjiangyuan region of China. The assembly and annotation of the developmental transcriptome of SDM performed in this study revealed tissue-specific gene expression patterns and pathways. This global analysis of the spruce dwarf mistletoe transcriptome revealed candidate genes that can be further characterized to increase our knowledge of the physiological development of *Arceuthobium*, bringing us one step closer to uncovering the molecular mechanisms underlying the development and plant parasitism of spruce dwarf mistletoe.

## Additional files

Additional file 1:
**Table S1.** Primers used in RT-PCR analysis. (XLS 27 kb) Additional file 2:
**Figure S1.** RT-PCR validation of RNA-seq results. Validation of the presence of annotated transcripts as detected by RT-PCR in cDNA of SDM. Lanes 1–6 stands for the predicted unigenes such as c89665_g2, c128315_g1, c139652_g1, c142396_g5, c138012_g1 and c140166_g1. (TIF 1056 kb) Additional file 3:
**Table S2.** List of tissue-specific genes. (XLS 4063 kb) Additional file 4
**Table S3.** The list of all up/down-regulated DEGs in reproductive compared with vegetative stage. (XLS 5338 kb) Additional file 5:
**Figure S2.** qRT PCR analysis of the putative unigenes. The Y axis represents relative expression of genes as obtained by the △△CT method. (TIF 27134 kb) Additional file 6:
**Table S4.** Expression profiles of putative transcription factors in different developmental tissues. (XLS 416 kb) Additional file 7:
**Table S5.** Expression profiles of putative protein kinases in different developmental tissues. (XLS 2842 kb) Additional file 8:
**Table S6.** Expression profiles of putative transporters in different developmental tissues. (XLS 2893 kb) Additional file 9:
**Table S7.** Expression profiles of putative plant hormone-associated genes in different developmental tissues. (XLS 682 kb) Additional file 10:
**Figure S3.** Heatmap diagrams of relative expression levels of DEGs annotated in signal transduction (A), plat hormone (B), transcription factors (C), biogenesis (D) and transporters (E). (TIF 2526 kb) Additional file 11:
**Table S8.** Differential expression of gene involved in carbohydrate metabolism and energy (XLS 1023 kb) Additional file 12:
**Table S9.** Expression profiles of putative unigenes related to photosynthesis in different developmental tissues. (XLS 170 kb) Additional file 13:
**Table S10.** GO enrichment of DGE. (XLS 115 kb)

## Abbreviations

DEG: Differentially expressed genes
DMR: Dwarf mistletoe rating
FPKM: Fragments per kilobase per transcript per million mapped reads
KO: KEGG ortholog database
KOG: Eukaryotic ortholog groups database
ᅟ: Next generation sequencing
NR: NCBI nonredundant database
NT: NCBI nucleotide sequences database
PFAM: Protein family
GO: Gene Ontology
RIN: RNA integrity number
RSEM: RNA-seq by expectation maximization
SDM: Spruce dwarf mistletoe
