# Supplementary figures and images for: De novo assembly and transcriptome characterization of spruce dwarf mistletoe Arceuthobium sichuanense uncovers gene expression profiling associated with plant development
Source: BMC Genomics. 2016 Oct 1;17:771. doi: 10.1186/s12864-016-3127-y (PMC5045590; doi:10.1186/s12864-016-3127-y)

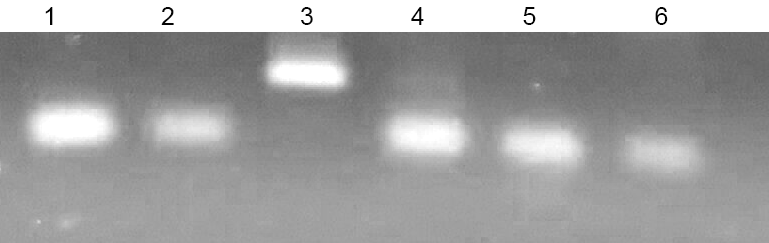

Supplement: Additional file 2: — Figure S1. RT-PCR validation of RNA-seq results. Validation of the presence of annotated transcripts as detected by RT-PCR in cDNA of SDM. Lanes 1–6 stands for the predicted unigenes such as c89665_g2, c128315_g1, c139652_g1, c142396_g5, c138012_g1 and c140166_g1. (TIF 1056 kb) [file 12864_2016_3127_MOESM2_ESM.tif]

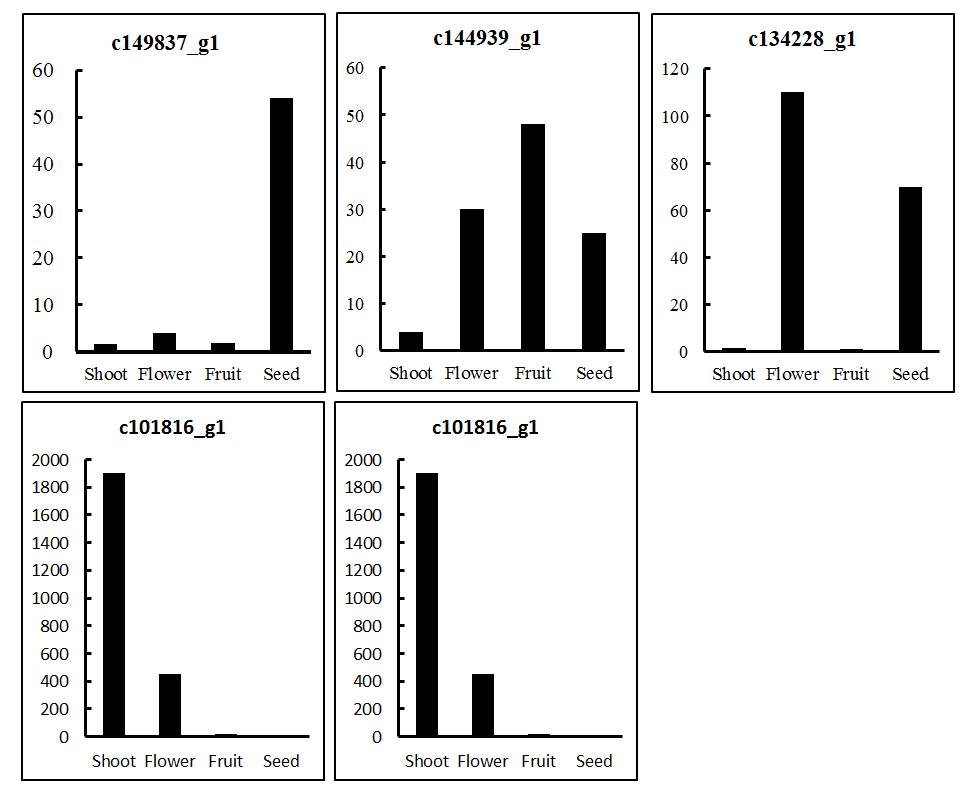

Supplement: Additional file 5: — Figure S2. qRT PCR analysis of the putative unigenes. The Y axis represents relative expression of genes as obtained by the △△CT method. (TIF 27134 kb) [file 12864_2016_3127_MOESM5_ESM.tif]

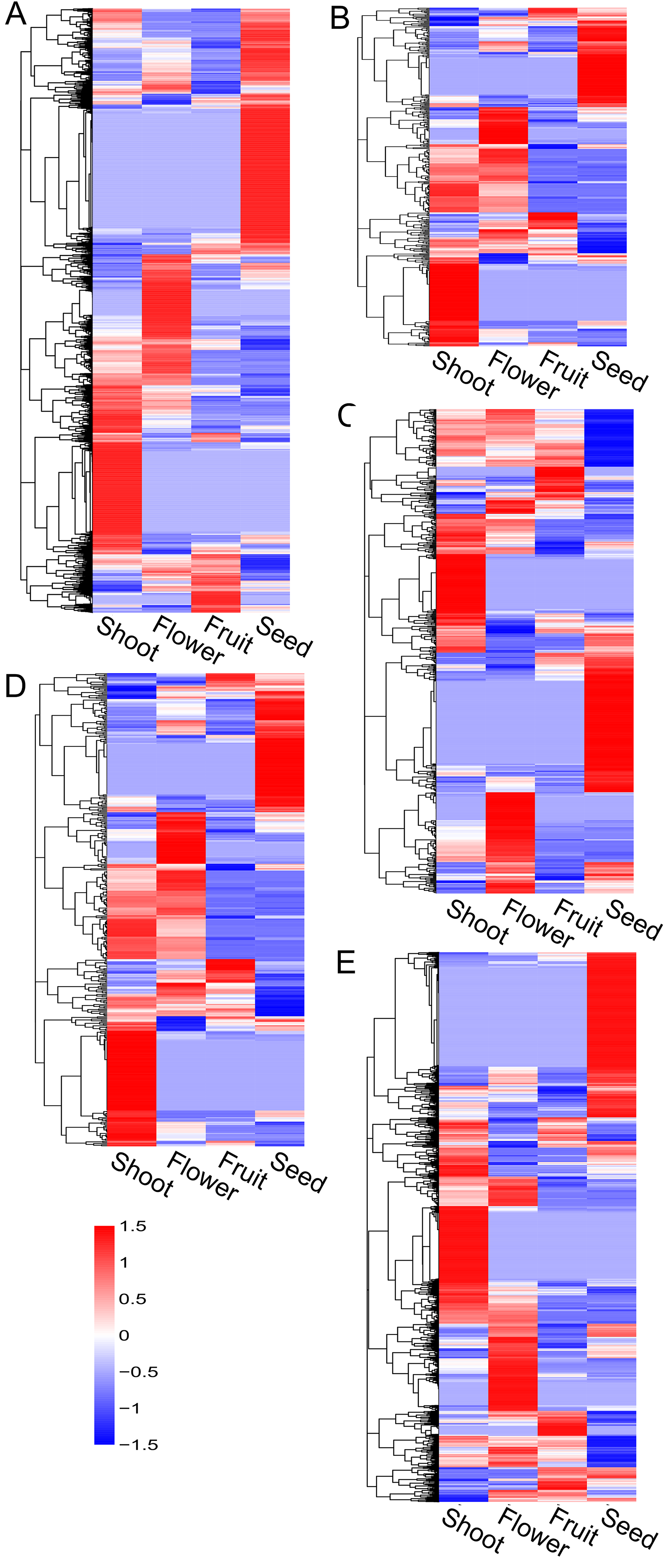

Supplement: Additional file 10: — Figure S3. Heatmap diagrams of relative expression levels of DEGs annotated in signal transduction (A), plat hormone (B), transcription factors (C), biogenesis (D) and transporters (E). (TIF 2526 kb) [file 12864_2016_3127_MOESM10_ESM.tif]
